# Body mass index, neck circumference, and hypertension: a prospective cohort study

**DOI:** 10.3389/fcvm.2023.1269328

**Published:** 2023-10-02

**Authors:** Tao-jun Ren, Kun Zhang, Wen-juan Li, Shu-tang Ren, Yun-zhou Huang, Ning Yang, Shou-ling Wu, Yu-ming Li

**Affiliations:** ^1^ Clinical School of Cardiovascular Disease, Tianjin Medical University, Tianjin, China; ^2^ Department of Cardiology, TEDA International Cardiovascular Hospital, Tianjin, China; ^3^ Graduate School, North China University of Science and Technology, Tangshan, China; ^4^ Department of Cardiology, Kailuan General Hospital, Tangshan, China

**Keywords:** BMI, neck circumference, hypertension, kailuan, prospective cohort study

## Abstract

**Objective:**

This study aimed to investigate the association between BMI combined with neck circumference and the risk of hypertension.

**Methods:**

We selected participants from the Kailuan study in 2014 who were normotensive as our research subjects. We compared the risk of hypertension among individuals in group 1 (non-obese with low neck circumference), group 2 (non-obese with high neck circumference), group 3 (obese with low neck circumference), and group 4 (obese with high neck circumference).

**Results:**

After a median observation period of 3.86 years, hypertension occurred in 13,383 participants. Subjects in Group 2, 3, and 4 had significantly higher risks of hypertension compared to Group 1, with hazard ratios (HRs) of 1.066 (95% CI: 1.025, 1.110), 1.322 (95% CI: 1.235, 1.415), and 1.422 (95% CI: 1.337, 1.512), respectively. Additionally, adding BMI to a conventional model had a greater incremental effect on predicting hypertension compared to adding neck circumference alone. However, considering both BMI and neck circumference together further improved the prediction of hypertension.

**Conclusion:**

Individuals with both high BMI and high neck circumference face a higher risk of hypertension. Moreover, BMI is a superior predictor of hypertension risk compared to neck circumference, but using both of these measures can further enhance the accuracy of hypertension risk prediction.

## Introduction

Hypertension is a significant global public health challenge characterized by high prevalence and disability rates. In 2019, the World Health Organization reported an age-standardized prevalence of hypertension in adults aged 30–79 years worldwide, with rates of 34% in men and 32% in women (1). In China, the prevalence of hypertension has continued to rise over the past three decades due to factors such as population aging and unhealthy lifestyles. The prevalence of hypertension (≥140/90 mmHg) surged from 11.3% in 1991 (2) to 18.8% in 2002 (3), and to 23.2% in 2012–2015 (4). Consequently, hypertension remains a significant health concern in China. Hypertension can lead to multiple organ damage and increase the risk of cardiovascular events, renal diseases, and cerebrovascular accidents. It is crucial to identify individuals at high risk of developing hypertension early to prevent and manage this condition effectively.

Risk factors for hypertension include high-sodium and low-potassium diets, physical inactivity, psychosocial stress, smoking, alcohol consumption, lack of sleep, and obesity (5). Obesity, often assessed by body mass index (BMI), is a well-established risk factor for elevated blood pressure, and obese individuals are at higher risk of developing hypertension than those with a normal BMI (6). Neck circumference, a novel measure of central obesity, has also been found to be positively correlated with blood pressure (7, 8). However, there is a scarcity of prospective studies investigating the association between neck circumference and hypertension. To our knowledge, no studies have determined whether BMI or neck circumference is the superior predictor of hypertension risk or if their combined use enhances predictive accuracy. Therefore, this prospective cohort study aimed to investigate the roles of BMI and neck circumference in estimating the future risk of hypertension in the Kailuan population in Hebei, China.

## Methods

### Study design and study population

The Kailuan study is a community-based research project conducted in Tangshan, a city in northern China. The initial physical examination of employed and retired Kailuan Group employees at Kailuan General Hospital and its 11 affiliated hospitals took place in 2006–2007 (FY2006), with follow-up visits every two years. The 5th follow-up visit (FY 2014) introduced the measurement of neck circumference. For this study, we selected participants from the 2014 health examination as research subjects.

**Figure 1:**
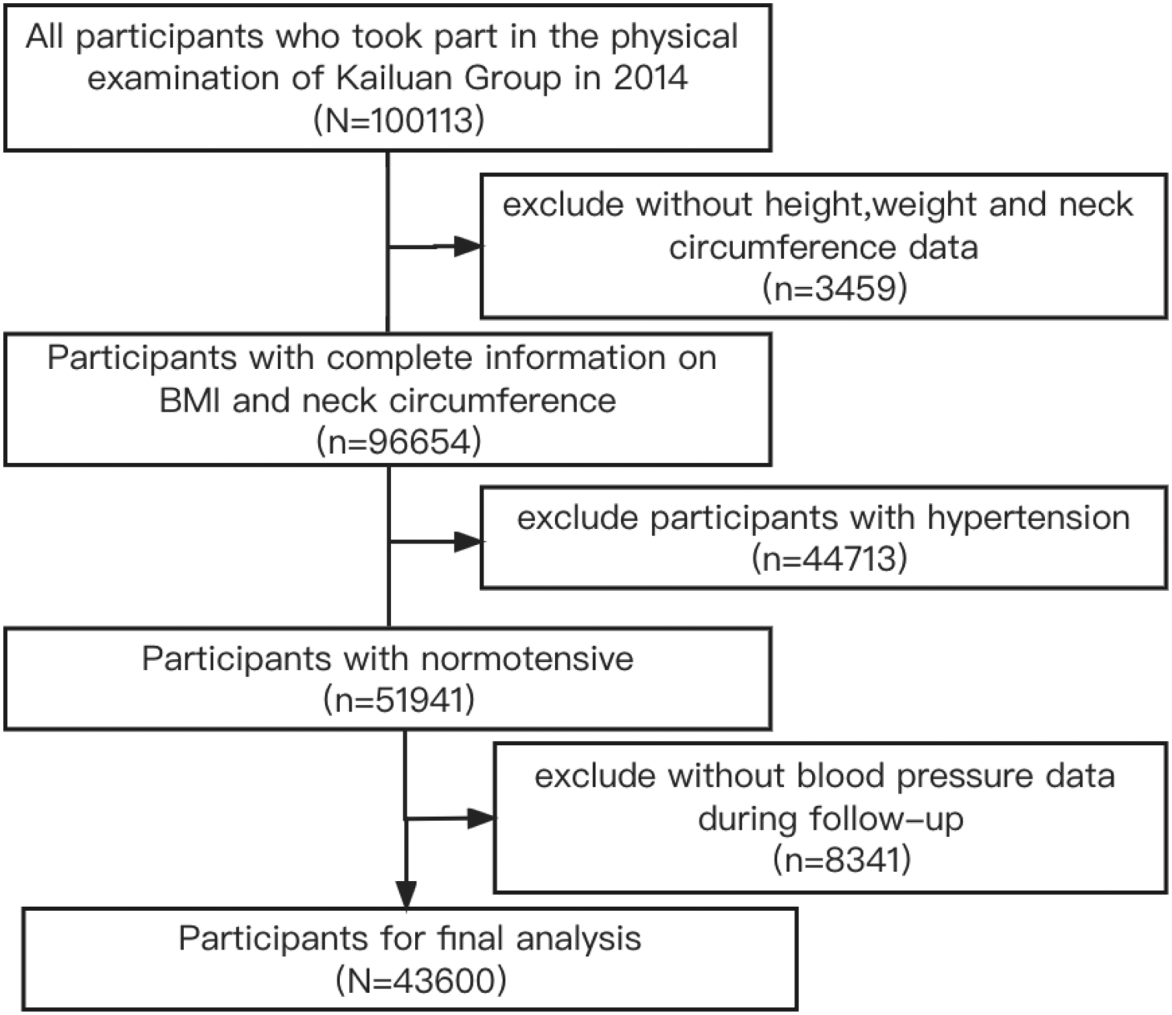
Flow chart of inclusion and exclusion.

Inclusion criteria were as follows: (1) participation in the 2014 health examination; (2) complete information on BMI and neck circumference; (3) agreement to participate in this study and signing of informed consent. Exclusion criteria included: (1) participants with hypertension in 2014; (2) lack of blood pressure data during follow-up. The flow chart is presented in Figure 1. This study adhered to the Declaration of Helsinki and received approval from the Ethics Committee of Kailuan General Hospital (200605).

### Data collection

#### BMI measurement

Height was measured without shoes or hats with an accuracy of 0.1 cm, and weight was measured using calibrated platform scales with an accuracy of 0.1 kg. BMI was calculated as body mass (kg) divided by height squared (m^2^). A BMI ≥ 28 kg/m^2^ was defined as obesity.

#### Neck circumference measurement

Subjects were measured while sitting, looking straight ahead, and breathing calmly. A soft ruler was positioned directly below the thyroid cartilage to measure neck circumference, with an accuracy of 0.1 cm (9).

#### BP measurement

Blood pressure was measured in a consultation room by uniformly trained and qualified medical personnel using an Omron HEM-8102A electronic sphygmomanometer. Before measuring blood pressure, subjects were required to sit and rest quietly for at least 5 min, and they were not allowed to smoke or consume coffee or tea within 30 min. Sitting blood pressure was assessed thrice with intervals of 1–2 min, and the resulting average value was documented as the measurement outcome. Hypertension was characterized by an in-office systolic blood pressure of ≥140 mmHg and/or a diastolic blood pressure of ≥90 mmHg, a previous history of hypertension, or the utilization of antihypertensive medication (10).

### Assessment of covariates

Basic information on the subjects was collected using questionnaires, including age, gender, smoking, drinking, salt intake, physical activity, education level, and nighttime sleep duration. Salt intake was assessed through a questionnaire survey, and participants categorized their daily habitual salt intake as low, moderate, or high. Previous cohort studies have established that 24-h urinary sodium excretion levels corresponding to low, moderate, and high salt intake are <6 g, 6–10 g, and >10 g, respectively (11). Physical activity was classified as never, occasional, or frequent, while education level was categorized as junior high school or below and high school or above. Additionally, information on diabetes history, antidiabetic treatment, lipid-lowering treatment, cardiovascular diseases (CVD), and cancer was obtained through questionnaires. Diabetes mellitus was defined as fasting blood glucose ≥7.0 mmol/L, a history of diabetes mellitus, or the use of antidiabetic therapy (12). CVD types included myocardial infarction (MI), ischemic stroke (IS), and hemorrhagic stroke (HS).

Laboratory test parameters were acquired by drawing venous blood from fasting individuals during the day of the physical examination. Fasting blood glucose, total cholesterol, creatinine, and high-sensitivity C-reactive protein levels were assessed using a Hitachi 7,600 Automatic Analyzer. Estimated glomerular filtration rate (eGFR) was estimated using the chronic kidney disease epidemiology collaboration (CKD-EPI) equation.

### Grouping

Participants can be categorized based on BMI into non-obese and obese cohorts (13) (BMI < 28 and ≥28 kg/m^2^, respectively) or by neck circumference into low and high neck circumference cohorts (neck circumference <40 and ≥40 cm, respectively) following the 75th percentile of neck circumference and related references (14, 15). Consequently, the participants were divided into four groups according to BMI and neck circumference: group 1—non-obese and low neck circumference (BMI < 28 kg/m^2^ and neck circumference <40 cm); group 2—non-obese and high neck circumference (BMI < 28 kg/m^2^ and neck circumference ≥40 cm); group 3—obese and low neck circumference (BMI ≥ 28 kg/m^2^ and neck circumference <40 cm); and group 4—obese and high neck circumference (BMI ≥ 28 kg/m^2^ and neck circumference ≥40 cm).

### Cohort follow-up and outcome

The follow-up started at the time of completing the baseline (2014) physical examination, and the occurrence of hypertension was considered the endpoint event. The last physical examination time without the endpoint event marked the end of follow-up. The median follow-up period was 3.86 years.

### Statistical analysis

Measurement data with a normal distribution were presented as mean ± standard deviation (*x* ± s), and analysis of variance was used for comparing different groups. Non-normally distributed measurement data were expressed as a median and interquartile range, and the Kruskal-Wallis rank-sum test was used for comparison. Enumeration data groups were expressed as numbers (%) and compared using the chi-square test. The cumulative incidence of endpoint events in different groups was calculated using the Kaplan-Meier method, and differences among groups were assessed with the log-rank test. The incidence densities of new-onset hypertension between different groups were calculated by dividing the number of events by the total person-years of follow-up (per 1,000/person-year). We evaluated the interaction between BMI and neck circumference on the risk of hypertension. To analyze the effects of new-onset hypertension in different groups, Cox proportional hazards models were employed. Model 1 was adjusted for age and sex; model 2 was further adjusted for education, salt intake, smoking, drinking, physical activity, total cholesterol, estimated glomerular filtration rate, high-sensitivity C-reactive protein, nighttime sleep duration, diabetes, antidiabetic treatment, and lipid-lowering treatment; model 3 was further adjusted for baseline systolic blood pressure based on model 2.

To ensure the robustness of our findings, several sensitivity analyses were performed: (1) BMI was categorized into two levels according to the Asian standards (non-obese: BMI < 25 kg/m^2^, obese: BMI ≥ 25 kg/m^2^) (16). (2) Participants with diabetes were excluded. (3) Those using antidiabetic treatment or lipid-lowering treatment were excluded. (4) Cut-off of neck circumference in the prediction of hypertension using ROC curve and considering sex heterogeneity was explored. Further, neck circumference was categorized into two levels according to cut-off point by using ROC curve. (5) Those with CVD were excluded. (6) Participants with cancer were also excluded. Additionally, stratified analyses were conducted to analyze the effects of BMI and neck circumference on hypertension in different subgroups based on age (<60 y, ≥60 y), sex (male, female), and nighttime sleep duration (<7 h, ≥7 h). To assess the predictive ability of different models for hypertension risk, including the traditional model + BMI, traditional model + neck circumference, and traditional model + BMI + neck circumference, C statistics, net reclassification improvement index (NRI), and integrated discrimination improvement index (IDI) were calculated. The *p*-value was corrected for multiple testing using the Bonferroni correction. Statistical analyses were performed using SAS 9.4 and R 4.2.1 software, with *p* < 0.05 (two-sided) considered statistically significant.

## Results

### Baseline characteristics

In total, 10,113 individuals participated in the 2014 health examination of the Kailuan Group. Among them, 96,654 individuals had complete data for neck circumference and BMI, with 44,713 having hypertension and 51,941 having normal blood pressure. Of those with normal blood pressure, 8,341 with no blood pressure data during follow-up were excluded. A comparison of baseline characteristics between the loss-to-follow-up participants and the remaining participants is provided in Supplementary Table S1. Ultimately, 43,600 participants underwent statistical analysis, including 32,765 men (75.15%), with an average age of 46.19 ± 13.50 years. Systolic blood pressure, diastolic blood pressure, BMI, neck circumference, fasting blood glucose, total cholesterol, eGFR and high-sensitivity C-reactive protein were higher in group 4 than in group 1, along with a higher proportion of men, smokers, alcohol consumers, preference for saltiness, diabetes, and CVD, all with statistically significant differences (Table 1).

**Table 1:** Baseline characteristics of the study population (*N* = 43,600).

	Total	Group 1	Group 2	Group 3	Group 4	*P* value
	(*N* = 43,600)	(*N* = 26,408)	(*N* = 11,904)	(*N* = 2,499)	(*N* = 2,789)	
Age, year	46.19 ± 13.50	46.02 ± 13.64	47.40 ± 13.21	45.96 ± 13.18	42.89 ± 12.96	<0.001
Male, *N* (%)	32,765 (75.15)	17,667 (66.90)	10,957 (92.04)	1,602 (64.11)	2,539 (91.04)	<0.001
SBP, mmHg	122.31 ± 10.66	121.06 ± 10.98	123.70 ± 9.90	124.52 ± 10.01	126.25 ± 9.09	<0.001
DBP, mmHg	76.21 ± 7.58	75.22 ± 7.70	77.43 ± 7.13	77.61 ± 7.36	79.02 ± 6.75	<0.001
BMI, kg/m^2^	24.32 ± 3.32	23.20 ± 2.48	24.18 ± 2.33	30.15 ± 2.88	30.34 ± 2.36	<0.001
NC, cm	38.06 ± 2.79	36.52 ± 2.13	40.92 ± 1.13	37.19 ± 1.94	41.27 ± 1.30	<0.001
FBG, mmol/L	5.44 ± 1.33	5.36 ± 1.23	5.50 ± 1.45	5.64 ± 1.54	5.66 ± 1.47	<0.001
TC, mmol/L	5.03 ± 0.99	5.00 ± 1.00	5.03 ± 0.95	5.17 ± 0.99	5.15 ± 0.99	<0.001
eGFR, ml/min/1.73 m^2^	107.57 (90.51,124.88)	106.27 (89.54,122.54)	111.06 (92.87,131.31)	104.99 (89.41,120.05)	110.10 (91.25,130.97)	<0.001
hs-CRP, mg/L	0.99 (0.43,2.00)	0.92 (0.40,1.90)	0.90 (0.43,1.80)	1.40 (0.67,2.93)	1.44 (0.70,2.96)	<0.001
Nighttime sleep duration, h	6.68 ± 1.55	6.66 ± 1.58	6.74 ± 1.44	6.62 ± 1.60	6.63 ± 1.64	0.022
Smoking, *N* (%)	17,790 (40.80)	9,492 (35.94)	6,057 (50.88)	842 (33.69)	1,399 (50.16)	<0.001
Drinking, *N* (%)	20,369 (46.72)	11,777 (44.60)	6,081 (51.08)	1,075 (43.02)	1,436 (51.49)	<0.001
Salt status, *N* (%)						<0.001
Low salt	5,849 (13.42)	3,856 (14.60)	1,350 (11.34)	313 (12.53)	330 (11.83)	
Moderate salt	34,226 (78.50)	20,421 (77.33)	9,700 (81.49)	1,943 (77.75)	2,162 (77.52)	
High salt	3,525 (8.08)	2,131 (8.07)	854 (7.17)	243 (9.72)	297 (10.65)	
High school or above, *N* (%)	15,339 (35.18)	9,786 (37.06)	3,570 (29.99)	901 (36.05)	1,082 (38.80)	<0.001
Physical activity, *N* (%)						<0.001
Never	11,572 (26.54)	7,562 (28.64)	2,605 (21.88)	716 (28.65)	689 (24.70)	
Occasionally	28,207 (64.69)	16,384 (62.04)	8,366 (70.28)	1,562 (62.51)	1,895 (67.95)	
Frequent	3,821 (8.76)	2,462 (9.32)	933 (7.84)	221 (8.84)	205 (7.35)	
Diabetes, *N* (%)	3,234 (7.42)	1,624 (6.15)	1,050 (8.82)	263 (10.52)	297 (10.65)	<0.001
CVD, *N* (%)	1,021 (2.34)	548 (2.08)	326 (2.74)	62 (2.48)	85 (3.05)	<0.001
Cancer, *N* (%)	452 (1.04)	291 (1.10)	113 (0.95)	30 (1.20)	18 (0.65)	0.079
Antidiabetic treatment, *N* (%)	1,192 (2.73)	612 (2.32)	396 (3.33)	93 (3.72)	91 (3.26)	<0.001
Lipid-lowering treatment, *N* (%)	1,401(3.21)	714(2.70)	453(3.81)	95(3.80)	139(4.98)	<0.001

SBP, systolic blood pressure; DBP, diastolic blood pressure; BMI, body mass index; NC, neck circumference; FBG, fasting blood glucose; TC, total cholesterol; eGFR, estimated glomerular filtration rate;hs-CRP, high-sensitivity C reactive protein; CVD, cardiovascular diseases.

### Risk of hypertension in groups by BMI and neck circumference

**Figure 2:**
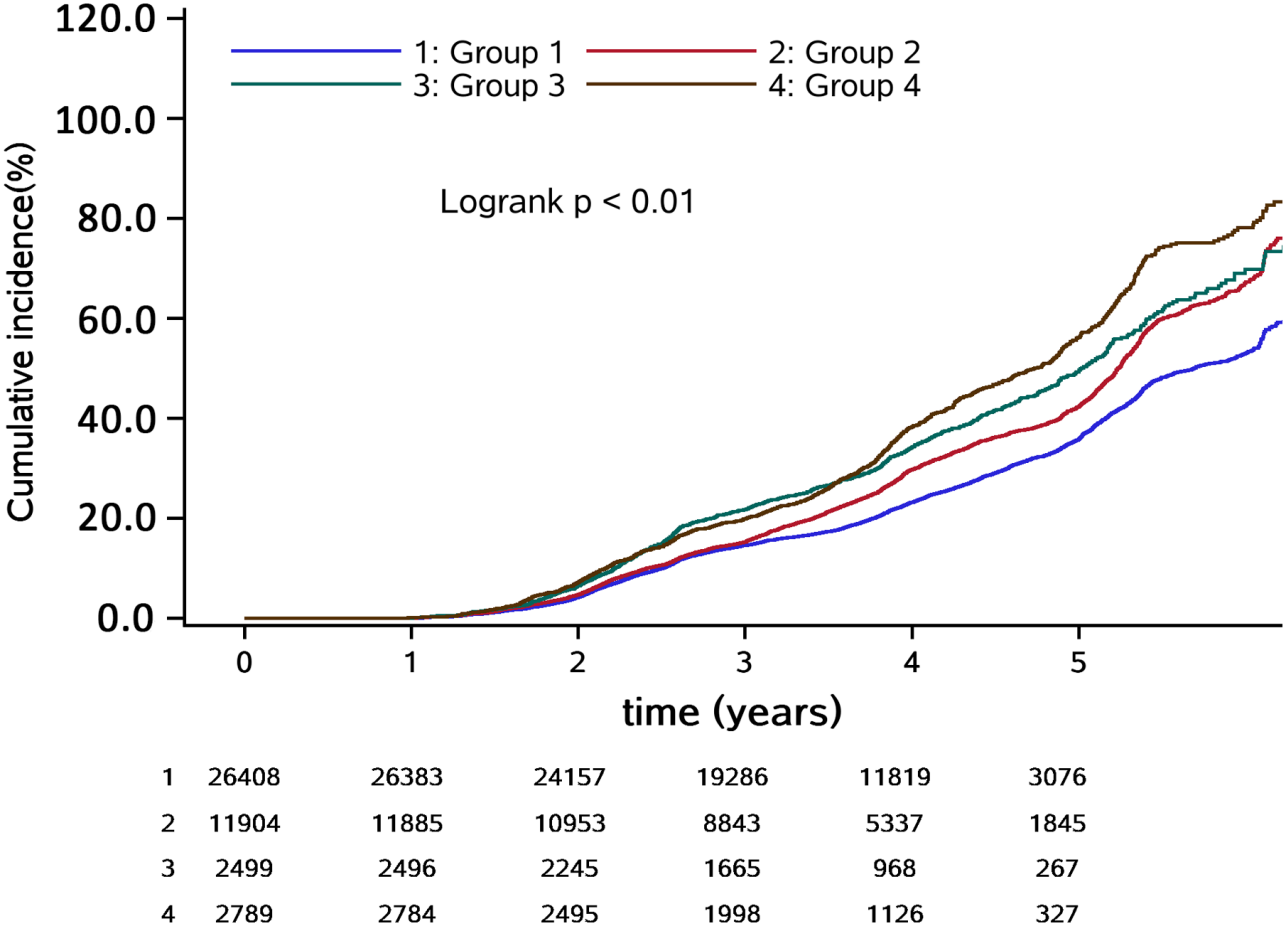
Cumulative incidence of hypertension.

The median follow-up period was 3.86 years, during which 13,383 participants (30.69%) developed hypertension. The incidence densities of groups 1, 2, 3, and 4 were 71.82, 92.43, 107.23, and 123.84 per thousand person-years, respectively (Table 2). The cumulative incidence of hypertension among the different groups was statistically significant based on the log-rank test (*P* < 0.05) (Figure 2).

**Table 2:** Association of different BMI and NC with risk of hypertension (*N* = 43,600).

	Group 1	Group 2	Group 3	Group 4	*P* for trend
*N* (%)	7,061 (26.74)	4,111 (34.53)	955 (38.22)	1,256 (45.03)	
Incidence, per 1,000 person-years	71.82	92.43	107.23	123.84	
Model 1	1.000	1.285 (1.236,1.335)	1.550 (1.449,1.658)	1.784 (1.680,1.894)	<0.001
Model 2	1.000	1.115 (1.072,1.160)	1.593 (1.489,1.704)	1.740 (1.637,1.849)	<0.001
Model 3	1.000	1.107 (1.064,1.152)	1.540 (1.439,1.648)	1.669 (1.570,1.774)	<0.001
Model 4	1.000	1.066 (1.025,1.110)	1.322 (1.235,1.415)	1.422 (1.337,1.512)	<0.001

Model 1: unadjusted; Model 2: Adjusted for age and gender; Model 3: Further adjusted for Education level, Salt status, Smoking, Drinking, Physical activity, TC, eGFR, hs-CRP, nighttime sleep duration, Diabetes, Antidiabetic treatment, Lipid-lowering treatment; Model 4: Further adjusted for SBP at baseline.

**Figure 3:**
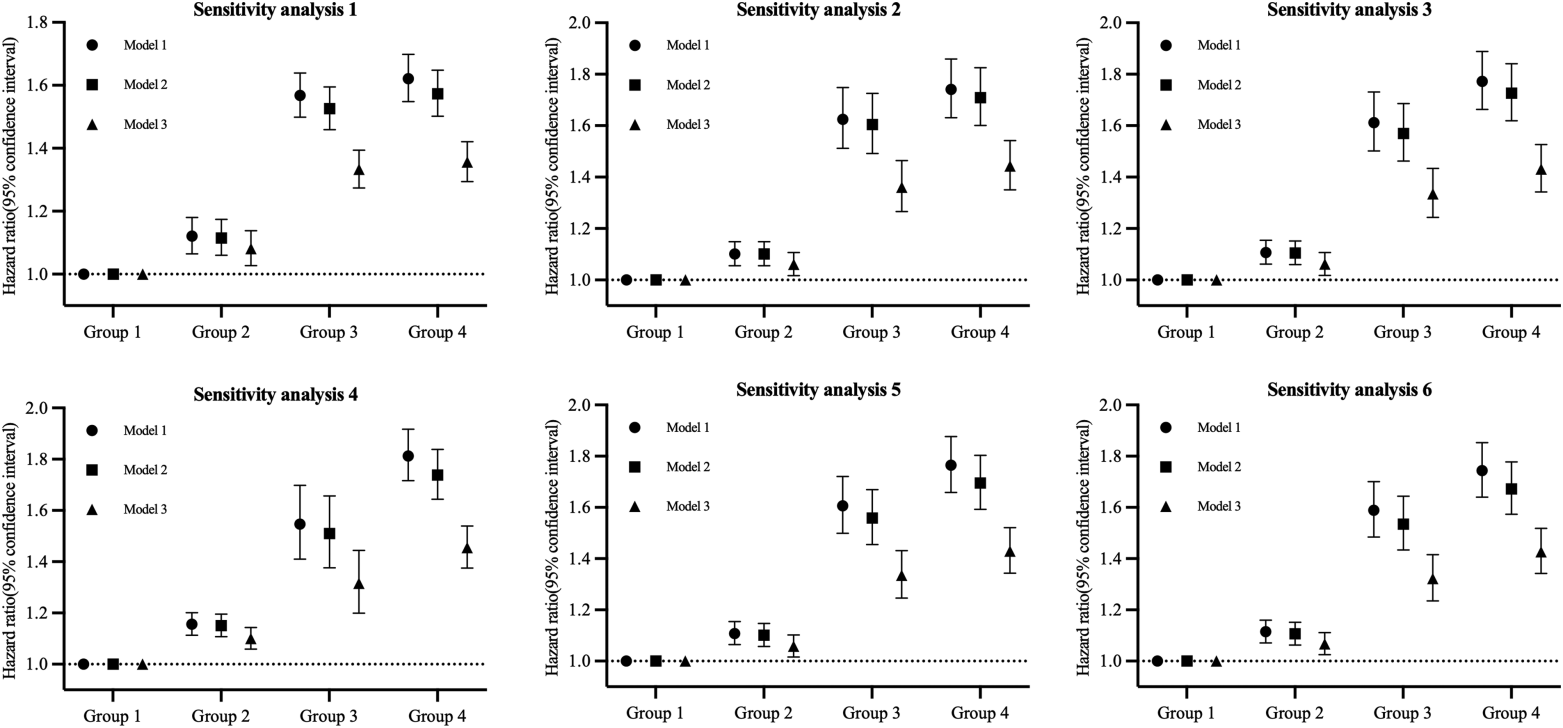
Sensitivity analyses for the association of BMI and NC with the risk of hypertension.

We observed no significant multiplicative or additive interaction between BMI and neck circumference [*P* for additive interaction >0.05; RERI = 0.031 (95% CI: −0.051, 0.113); *P* for multiplicative interaction >0.05]. After adjusting for potential confounders, the risk of hypertension gradually increased in groups 2, 3, and 4 in comparison to group 1, with hazard ratios (HRs) [95% confidence interval (CI)] of 1.066 (1.025, 1.110), 1.322 (1.235, 1.415), and 1.422 (1.337, 1.512), respectively. The trend was statistically significant with *P* < 0.001 (Table 2). Sensitivity analyses yielded results consistent with the main findings (Figure 3, Supplementary Tables S2–S7).

Additionally, we separately examined the relationship between different BMI and neck circumference groups and the risk of hypertension. The results indicated that elevated BMI and neck circumference levels increased the risk of hypertension (Tables 3, 4). Sensitivity analyses supported the main results (Supplementary Tables S8–S17).

**Table 3:** Association between BMI and risk of hypertension (*N* = 43,600).

	BMI < 24	BMI ≥ 24	*P* for trend
*N* (%)	11,172 (29.16)	2,211 (41.81)	
Incidence, per 1,000 person-years	78.24	116.08	
Model 1	1.000	1.609 (1.537,1.685)	<0.001
Model 2	1.000	1.522 (1.453,1.595)	<0.001
Model 3	1.000	1.328 (1.267,1.392)	<0.001

Model 1:Adjusted for age and gender; Model 2: Further adjusted for Education level, Salt status, Smoking, Drinking, Physical activity, TC, high neck circumference, eGFR, hs-CRP, nighttime sleep duration, Diabetes, Antidiabetic treatment, Lipid-lowering treatment; Model 3:Further adjusted for SBP at baseline.

**Table 4:** Association between NC and risk of hypertension (*N* = 43,600).

	Low neck circumference	High neck circumference	*P* for trend
*N* (%)	8,016 (27.73)	5,367 (36.53)	
Incidence per 1,000 person-years	74.77	98.26	
Model 1	1.000	1.166 (1.125,1.209)	<0.001
Model 2	1.000	1.103 (1.064,1.144)	<0.001
Model 3	1.000	1.068 (1.030,1.108)	<0.001

Model 1: Adjusted for age and gender; Model 2: Further adjusted for Education level, Salt status, Smoking, Drinking, Physical activity, obesity, TC, eGFR, hs-CRP, nighttime sleep duration, Diabetes, Antidiabetic treatment, Lipid-lowering treatment; Model 3: Further adjusted for SBP at baseline.

Sensitivity analysis 1 used 25 kg/m^2^ as a cut-off point to redefine obesity. Sensitivity analysis 2 was performed by excluding participants with diabetes. Sensitivity analysis 3 was performed by excluding participants on antidiabetic treatment and lipid-lowering treatment. Sensitivity analysis 4 used 38.25 cm (men) and 36 cm (women) as cut-off points to redefine high neck circumference. Sensitivity analysis 5 involved the exclusion of participants with CVD. Sensitivity analysis 6 was conducted by excluding participants with cancer.

Model 1: Adjusted for age and gender; Model 2: Further adjustments included Education level, Salt status, Smoking, Drinking, Physical activity, TC, eGFR, hs-CRP, nighttime sleep duration, Diabetes, Antidiabetic treatment, Lipid-lowering treatment; Model 3: Additional adjustment incorporated SBP at baseline.

### Stratified analysis

**Figure 4:**
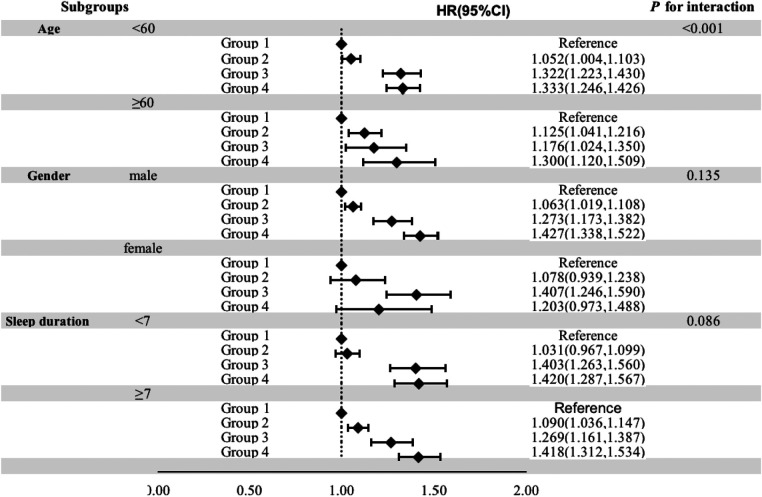
Stratified analysis for the association of BMI and NC with the risk of hypertension.

The stratified analysis demonstrated that groups were significantly associated with the risk of hypertension in participants younger than 60 years, male sex, and those whose nighttime sleep duration was less than 7 h, with HRs (95% CI) of 1.333 (1.246, 1.426), 1.427 (1.338, 1.522), and 1.420 (1.287, 1.567) in group 4, respectively (Figure 4).

### Predictive value of BMI and neck circumference

We compared the predictive value of BMI and neck circumference for the risk of hypertension (Table 5). The C statistics of the conventional model significantly improved with the incorporation of BMI (from 0.6699 to 0.6738, *P* < 0.001) and neck circumference (from 0.6699 to 0.6714, *P* < 0.001). The improvement was more significant after the incorporation of BMI. NRI improved more significantly after the incorporation of neck circumference (19.10%, *P* < 0.001) than that of BMI (12.93%, *P* < 0.001), and IDI improved more significantly after incorporating BMI (0.23%, *P* < 0.001) than that of neck circumference (0.15%, *P* < 0.001). The C statistic increased to 0.6745 (*P* < 0.001), the NRI improved by 19.67% (*P* < 0.001), and the IDI increased by 0.33% (*P* < 0.001) when both BMI and neck circumference were incorporated into the traditional model, demonstrating significant enhancements compared to when each was included separately.

**Table 5:** Reclassification and discrimination statistics for BMI and NC.

	C statistics (95% CI)	NRI (95%CI),%	IDI (95%CI),%
Conventional model	0.6699 (0.6648,0.6751)	Reference	Reference
Conventional model + BMI	0.6738 (0.6687,0.6788), *P* < 0.001	12.93 (11.43,14.44), *P* < 0.001	0.23 (0.19,0.28), *P* < 0.001
Conventional model + NC	0.6714 (0.6663,0.6765), *P* < 0.001	19.10 (17.05,21.16), *P* < 0.001	0.15 (0.12,0.18), *P* < 0.001
Conventional model + BMI + NC	0.6745 (0.6695,0.6796), *P* < 0.001	19.67 (17.59,21.76), *P* < 0.001	0.33 (0.28,0.39), *P* < 0.001

NC indicates neck circumference, NRI, net reclassification index, and IDI integrated discrimination improvement.

Conventional model was adjusted for age, sex, Physical activity, Salt status, Education level, SBP, Diabetes, hypercholesterolemia, nighttime sleep duration.

## Discussion

An important finding of the present study was that not only high BMI alone and high neck circumference alone are risk factors for hypertension, but also individuals with both high BMI and high neck circumference have a higher risk of hypertension. Moreover, BMI is superior to neck circumference in predicting the risk of hypertension, but considering both of them can further improve the prediction of hypertension risk.

In our study, the risk of hypertension was 42.2% higher in both the high BMI and high neck circumference group compared to both the low BMI and low neck circumference group. It was also higher compared to the BMI-alone and neck circumference-alone groups (32.8% and 6.8%, respectively). This suggests that the association between hypertension risk and the combination of BMI and neck circumference is stronger than with a single index. While some studies have shown a positive correlation between BMI combined with neck circumference and the severity of obstructive sleep apnea-hypopnea syndrome (OSAHS), a leading cause of secondary hypertension, no previous research has analyzed the association between this combination and the risk of hypertension. Luo et al. (17) found that patients with severe OSAHS had the highest mean neck circumference and BMI, and the risk of OSAHS increased when BMI exceeded 25 kg/m^2^ and neck circumference exceeded 40 cm. Our study, in agreement with this, demonstrated that individuals with both high BMI and neck circumference face a higher risk of OSAHS and hypertension than those with low BMI and neck circumference. Hypertension risk can be influenced by factors such as diabetes (18), antidiabetic treatment (19), and lipid-lowering treatment (20). We conducted sensitivity analyses after categorizing participants by BMI according to Asian standards (non-obese: BMI < 25 kg/m^2^, obese: BMI ≥ 25 kg/m^2^) and excluding those with diabetes or receiving antidiabetic and lipid-lowering treatments. The results aligned with the main findings, indicating the robustness of our study's conclusions. These findings supplement previous research on the relationship between hypertension and BMI and neck circumference.

BMI is a well-established predictor of hypertension (21–24). Deng et al. (24) demonstrated that incorporating BMI into the traditional model increased the C-statistic from 0.679 to 0.692 and led to a 19% improvement in the net reclassification index. In our study, the C-statistic was 0.6738 when BMI was included in the traditional model, and it was 0.6714 when neck circumference replaced BMI. However, when both were included in the traditional model, the C-statistic increased to 0.6745. These results suggest that BMI is a better predictor of hypertension risk than neck circumference, but the combination of both BMI and neck circumference offers superior predictive value. Therefore, when assessing an individual's risk of developing hypertension, both BMI and neck circumference should be taken into consideration.

The distribution of body fat differs between genders, with males having longer pharynxes and more fat deposition in the upper airway, especially in cases of obesity. Consequently, the relationship between BMI, neck circumference, and hypertension risk may vary between males and females. Our stratified analyses revealed a significant association between the combination of BMI and neck circumference and hypertension risk in men but not in women. In contrast to our results, Fan S et al. (8) showed that neck circumference was associated with hypertension risk in both men and women, with a higher risk in men [odds ratio (OR) = 1.57, 95% CI 1.14–2.17] compared to women (OR = 1.51, 95% CI 1.20–1.90). While our study did not find a significant association between BMI combined with neck circumference and hypertension risk in women, it is essential to note that the smaller number of women in our cohorts may have affected the results.

Although this study identified an increased risk of hypertension associated with high BMI combined with high neck circumference, the specific mechanism could not be assessed due to the observational nature of the study. Previous research suggests several potential factors. Firstly, neck circumference serves as an indicator of subcutaneous fat distribution in the upper body, which is known to release a significant portion of free fatty acids into the bloodstream, particularly in individuals with obesity (25). Elevated free fatty acids can trigger the production of oxidative stress markers, leading to vascular endothelial damage and hypertension development (26–28). Secondly, studies have linked both BMI (29) and neck circumference (30) to sleep apnea-hypopnea syndrome (SAHS), a primary cause of secondary hypertension affecting 30% to 50% of hypertension patients (31). Lastly, obesity-related fat accumulation around the kidneys can physically compress them, resulting in the activation of the renin-angiotensin-aldosterone system, a mechanism leading to hypertension (32).

This study, conducted in a large community cohort with a substantial sample size, collected prospective data over a 3.86-year follow-up period to investigate the relationship between high BMI in combination with high neck circumference and the risk of hypertension. Furthermore, this study meticulously recorded participant information to uphold data quality standards. Finally, when compared to waist circumference, neck circumference exhibits excellent reliability among and within observers. In contrast to waist circumference, neck circumference remains unaffected by the timing of measurement and maintains consistency both before and after eating. This enhances the practicality and convenience of using neck circumference in winter and busy healthcare settings, ultimately reducing inconvenience for both patients and healthcare staff. Nevertheless, there are certain limitations to consider: Firstly, our study was conducted exclusively among employees of the Kailuan Group in Tangshan City, with a notable predominance of male participants. Therefore, further validation is needed to ascertain the generalizability of these findings to other populations. Second, though this study adjusted for many potential confounders, residual confounding unmeasured factors could not be completely excluded, such as environment and genes. Third, detailed data on other long-standing illnesses such as COPD, which may have influenced the future development of hypertension, were not collected. Fourth, additional research is required to explore the prolonged impacts of fluctuating BMI and neck circumference on the progression of blood pressure. Fifth, the potential bias stemming from loss-to-follow-up may have impacted the subsequent development of hypertension.

## Conclusion

This study has illustrated a favorable correlation between the combination of BMI and neck circumference and the risk of hypertension. Furthermore, the predictive capacity for hypertension can be enhanced through the joint consideration of BMI and neck circumference. Consequently, in clinical practice, it is advisable to incorporate both BMI and neck circumference for a more effective assessment and prediction of hypertension risk, enabling timely and successful preventative measures.

## Data Availability

The raw data supporting the conclusions of this article will be made available by the authors, without undue reservation.
