# Supplementary material for: Body mass index, neck circumference, and hypertension: a prospective cohort study
Source: Front Cardiovasc Med. 2023 Oct 2;10:1269328. doi: 10.3389/fcvm.2023.1269328 (PMC10578437; doi:10.3389/fcvm.2023.1269328)
Supplement: Supplementary file 1 [file Table1.docx]

**Table S1. Characteristics of participants yes vs. no complete the follow-up surveys (N=51941)**

|  | complete the follow-up surveys | | *P* value |
| --- | --- | --- | --- |
|  | Yes (N=43600) | No (N=8341) |  |
| Age, year | 46.19±13.50 | 48.10±16.09 | <0.001 |
| Male, N(%) | 32765(75.15) | 7051(84.56) | <0.001 |
| SBP, mmHg | 122.31±10.66 | 122.82±10.35 | <0.001 |
| DBP, mmHg | 76.21±7.58 | 76.32±7.65 | 0.226 |
| BMI, kg/m² | 24.32±3.32 | 24.11±3.25 | <0.001 |
| NC, cm | 38.06±2.79 | 38.33±2.83 | <0.001 |
| FBG, mmol/L | 5.44±1.33 | 5.58±1.75 | <0.001 |
| TC, mmol/L | 5.03±0.99 | 5.01±1.65 | <0.001 |
| eGFR, ml/min /1.73m^2^ | 107.57  (90.51,124.88) | 104.89  (84.94,123.83) | 0.005 |
| hs-CRP, mg/L | 0.99(0.43,2.00) | 1.02(0.50,2.10) | <0.001 |
| nighttime sleep duration, h | 6.68±1.55 | 6.61±1.76 | 0.119 |
| Smoking, N(%) | 17790(40.80) | 3743(44.89) | <0.001 |
| Drinking, N(%) | 20369(46.72) | 3789(45.44) | 0.032 |
| Salt status, N(%) |  |  | 0.002 |
| Low salt | 5849(13.42) | 1174(14.08) |  |
| Moderate salt | 34226(78.50) | 6409(76.86) |  |
| High salt | 3525(8.08) | 755(9.05) |  |
| High school or above, N(%) | 15339(35.18) | 1953(23.42) | <0.001 |
| Physical activity, N(%) |  |  | <0.001 |
| Never | 11572(26.54) | 1869(22.42) |  |
| Occasionally | 28207(64.69) | 5712(68.51) |  |
| Frequent | 3821(8.76) | 757(9.08) |  |
| Diabetes, N(%) | 3234(7.42) | 767(9.20) | <0.001 |
| CVD, N(%) | 1021(2.34) | 305(3.66) | <0.001 |
| Cancer, N(%) | 452(1.04) | 123(1.48) | <0.001 |
| Antidiabetic treatment, N(%) | 1192(2.73) | 219(2.63) | 0.580 |
| Lipid-lowering treatment, N(%) | 1401(3.21) | 296(3.55) | 0.113 |

SBP, systolic blood pressure; DBP, diastolic blood pressure; BMI, body mass index; NC, neck circumference；FBG, fasting blood glucose; TC, total cholesterol; eGFR, estimated glomerular filtration rate ;hs-CRP, high-sensitivity C reactive protein; CVD, cardiovascular diseases

**Table S2. Association of Different BMI and NC With Risk of Hypertension using 25kg/m^2^ as cut-off point（N=43600）**

|  | Group 1 | Group 2 | Group 3 | Group 4 | *P* for trend |
| --- | --- | --- | --- | --- | --- |
| Model 1 | 1.000 | 1.121(1.065,1.180) | 1.568(1.499,1.639) | 1.621(1.548,1.698) | <0.001 |
| Model 2 | 1.000 | 1.115(1.060,1.174) | 1.526(1.459,1.595) | 1.573(1.502,1.648) | <0.001 |
| Model 3 | 1.000 | 1.081(1.027,1.138) | 1.333(1.274,1.394) | 1.356(1.294,1.421) | <0.001 |

Model 1：Adjusted for age and gender; Model 2：Further adjusted for Education level, Salt status, Smoking, Drinking , Physical activity, TC, eGFR, hs-CRP, nighttime sleep duration, Diabetes, Antidiabetic treatment, Lipid-lowering treatment; Model3：Further adjusted for SBP at baseline.

**Table S3. Association of Different BMI and NC With Risk of Hypertension by excluding diabetes（N=40366）**

|  | Group 1 | Group 2 | Group 3 | Group 4 | *P* for trend |
| --- | --- | --- | --- | --- | --- |
| Model 1 | 1.000 | 1.101(1.055,1.149) | 1.625(1.512,1.748) | 1.741(1.631,1.859) | <0.001 |
| Model 2 | 1.000 | 1.101(1.055,1.149) | 1.604(1.492,1.725) | 1.709(1.601,1.825) | <0.001 |
| Model 3 | 1.000 | 1.061(1.017,1.107) | 1.361(1.266,1.464) | 1.443(1.351,1.542) | <0.001 |

Model 1：Adjusted for age and gender; Model 2：Further adjusted for Education level, Salt status, Smoking, Drinking , Physical activity, TC, eGFR, hs-CRP, nighttime sleep duration, Lipid-lowering treatment; Model3：Further adjusted for SBP at baseline.

**Table S4. Association of Different BMI and NC With Risk of Hypertension by excluding Antidiabetic treatment and Lipid-lowering treatment（N=41295）**

|  | Group 1 | Group 2 | Group 3 | Group 4 | *P* for trend |
| --- | --- | --- | --- | --- | --- |
| Model 1 | 1.000 | 1.107(1.062,1.154) | 1.612(1.502,1.731) | 1.772(1.663,1.889) | <0.001 |
| Model 2 | 1.000 | 1.105(1.060,1.152) | 1.570(1.462,1.686) | 1.726(1.619,1.841) | <0.001 |
| Model 3 | 1.000 | 1.062(1.018,1.107) | 1.335(1.243,1.434) | 1.431(1.342,1.527) | <0.001 |

Model 1：Adjusted for age and gender; Model 2：Further adjusted for Education level, Salt status, Smoking, Drinking , Physical activity, TC, eGFR, hs-CRP, nighttime sleep duration, Diabetes; Model3：Further adjusted for SBP at baseline.

**Table S5. Association of Different BMI and NC With Risk of Hypertension using new NC cut-off point（N=43600）**

|  | Group 1 | Group 2 | Group 3 | Group 4 | *P* for trend |
| --- | --- | --- | --- | --- | --- |
| Model 1 | 1.000 | 1.156(1.113,1.201) | 1.547(1.410,1.698) | 1.813(1.716,1.917) | <0.001 |
| Model 2 | 1.000 | 1.151(1.108,1.196) | 1.510(1.376,1.657) | 1.738(1.644,1.838) | <0.001 |
| Model 3 | 1.000 | 1.100(1.059,1.143) | 1.316(1.199,1.444) | 1.455(1.375,1.539) | <0.001 |

Model 1：Adjusted for age and gender; Model 2：Further adjusted for Education level, Salt status, Smoking, Drinking , Physical activity, TC, eGFR, hs-CRP, nighttime sleep duration, Diabetes, Antidiabetic treatment, Lipid-lowering treatment; Model3：Further adjusted for SBP at baseline.

**Table S6. Association of Different BMI and NC With Risk of Hypertension by excluding CVD （N=42579）**

|  | Group 1 | Group 2 | Group 3 | Group 4 | *P* for trend |
| --- | --- | --- | --- | --- | --- |
| Model 1 | 1.000 | 1.108(1.065,1.154) | 1.606(1.499,1.721) | 1.765(1.659,1.877) | <0.001 |
| Model 2 | 1.000 | 1.101(1.057,1.147) | 1.559(1.455,1.670) | 1.695(1.593,1.803) | <0.001 |
| Model 3 | 1.000 | 1.058(1.016,1.102) | 1.335(1.246,1.431) | 1.429(1.343,1.521) | <0.001 |

Model 1：Adjusted for age and gender; Model 2：Further adjusted for Education level, Salt status, Smoking, Drinking , Physical activity, TC, eGFR, hs-CRP, nighttime sleep duration, Diabetes, Antidiabetic treatment, Lipid-lowering treatment; Model3：Further adjusted for SBP at baseline.

**Table S7. Association of Different BMI and NC With Risk of Hypertension by excluding cancer （N=43148）**

|  | Group 1 | Group 2 | Group 3 | Group 4 | *P* for trend |
| --- | --- | --- | --- | --- | --- |
| Model 1 | 1.000 | 1.115(1.071,1.160) | 1.589(1.484,1.701) | 1.744(1.640,1.853) | <0.001 |
| Model 2 | 1.000 | 1.107(1.063,1.152) | 1.535(1.434,1.644) | 1.672(1.573,1.778) | <0.001 |
| Model 3 | 1.000 | 1.067(1.025,1.111) | 1.322(1.235,1.416) | 1.427(1.342,1.518) | <0.001 |

Model 1：Adjusted for age and gender; Model 2：Further adjusted for Education level, Salt status, Smoking, Drinking , Physical activity, TC, eGFR, hs-CRP, nighttime sleep duration, Diabetes, Antidiabetic treatment, Lipid-lowering treatment; Model3：Further adjusted for SBP at baseline.

**Table S8. Association between BMI and risk of Hypertension using 25kg/m^2^ as cut-off point（N=43600）**

|  | BMI<25 | BMI≥25 | *P* for trend |
| --- | --- | --- | --- |
| Model 1 | 1.000 | 1.537(1.486,1.591) | <0.001 |
| Model 2 | 1.000 | 1.478(1.428,1.530) | <0.001 |
| Model 3 | 1.000 | 1.301(1.256,1.347) | <0.001 |

Model 1：Adjusted for age and gender; Model 2：Further adjusted for Education level, Salt status, Smoking, Drinking , Physical activity, TC, high neck circumference, eGFR, hs-CRP, nighttime sleep duration, Diabetes, Antidiabetic treatment, Lipid-lowering treatment; Model3：Further adjusted for SBP at baseline.

**Table S9 Association between BMI and risk of Hypertension by excluding diabetes（N=40366）**

|  | BMI<24 | BMI≥24 | *P* for trend |
| --- | --- | --- | --- |
| Model 1 | 1.000 | 1.632(1.554,1.714) | <0.001 |
| Model 2 | 1.000 | 1.576(1.500,1.657) | <0.001 |
| Model 3 | 1.000 | 1.361(1.294,1.431) | <0.001 |

Model 1：Adjusted for age and gender; Model 2：Further adjusted for Education level, Salt status, Smoking, Drinking , Physical activity, TC, high neck circumference, eGFR, hs-CRP, nighttime sleep duration, Lipid-lowering treatment; Model3：Further adjusted for SBP at baseline.

**Table S10. Association between BMI and risk of Hypertension by excluding Antidiabetic treatment and Lipid-lowering treatment（N=41295）**

|  | BMI<24 | BMI≥24 | *P* for trend |
| --- | --- | --- | --- |
| Model 1 | 1.000 | 1.639(1.562,1.720) | <0.001 |
| Model 2 | 1.000 | 1.566(1.491,1.644) | <0.001 |
| Model 3 | 1.000 | 1.342(1.278,1.410) | <0.001 |

Model 1：Adjusted for age and gender; Model 2：Further adjusted for Education level, Salt status, Smoking, Drinking , Physical activity, TC, high neck circumference, eGFR, hs-CRP, nighttime sleep duration, Diabetes; Model3：Further adjusted for SBP at baseline.

**Table S11 Association between BMI and risk of Hypertension by excluding CVD（N=42579）**

|  | BMI<24 | BMI≥24 | *P* for trend |
| --- | --- | --- | --- |
| Model 1 | 1.000 | 1.631(1.557,1.709) | <0.001 |
| Model 2 | 1.000 | 1,548(1.477,1.623) | <0.001 |
| Model 3 | 1.000 | 1.343(1.281,1.409) | <0.001 |

Model 1：Adjusted for age and gender; Model 2：Further adjusted for Education level, Salt status, Smoking, Drinking , Physical activity, TC, high neck circumference, eGFR, hs-CRP, nighttime sleep duration, Diabetes, Antidiabetic treatment, Lipid-lowering treatment; Model3：Further adjusted for SBP at baseline.

**Table S12 Association between BMI and risk of Hypertension by excluding cancer（N=43148）**

|  | BMI<24 | BMI≥24 | *P* for trend |
| --- | --- | --- | --- |
| Model 1 | 1.000 | 1.609(1.537,1.685) | <0.001 |
| Model 2 | 1.000 | 1.522(1.453,1.595) | <0.001 |
| Model 3 | 1.000 | 1.330(1.269,1.394) | <0.001 |

Model 1：Adjusted for age and gender; Model 2：Further adjusted for Education level, Salt status, Smoking, Drinking , Physical activity, TC, high neck circumference, eGFR, hs-CRP, nighttime sleep duration, Diabetes, Antidiabetic treatment, Lipid-lowering treatment; Model3：Further adjusted for SBP at baseline.

**Table S13. Association between NC and risk of Hypertension using new NC cut-off point（N=43600）**

|  | low neck circumference | high neck circumference | *P* for trend |
| --- | --- | --- | --- |
| Model 1 | 1.000 | 1.209(1.168,1.252) | <0.001 |
| Model 2 | 1.000 | 1.151(1.111,1.193) | <0.001 |
| Model 3 | 1.000 | 1.101(1.063,1.141) | <0.001 |

Model 1：Adjusted for age and gender; Model 2：Further adjusted for Education level, Salt status, Smoking, Drinking , Physical activity, obesity , TC, eGFR, hs-CRP, nighttime sleep duration, Diabetes, Antidiabetic treatment, Lipid-lowering treatment; Model3：Further adjusted for SBP at baseline.

**Table S14. Association between NC and risk of Hypertension by excluding diabetes（N=40366）**

|  | low neck circumference | high neck circumference | *P* for trend |
| --- | --- | --- | --- |
| Model 1 | 1.000 | 1.152(1.109,1.197) | <0.001 |
| Model 2 | 1.000 | 1.095(1.053,1.138) | <0.001 |
| Model 3 | 1.000 | 1.061(1.021,1.103) | <0.001 |

Model 1：Adjusted for age and gender; Model 2：Further adjusted for Education level, Salt status, Smoking, Drinking , Physical activity, obesity, TC, eGFR, hs-CRP, nighttime sleep duration, Lipid-lowering treatment; Model3：Further adjusted for SBP at baseline.

**Table S15. Association between NC and risk of Hypertension by excluding Antidiabetic treatment and Lipid-lowering treatment（N=41295）**

|  | low neck circumference | high neck circumference | *P* for trend |
| --- | --- | --- | --- |
| Model 1 | 1.000 | 1.162(1.120,1.207) | <0.001 |
| Model 2 | 1.000 | 1.104(1.063,1.147) | <0.001 |
| Model 3 | 1.000 | 1.064(1.024,1.105) | <0.001 |

Model 1：Adjusted for age and gender; Model 2：Further adjusted for Education level, Salt status, Smoking, Drinking , Physical activity, obesity, TC, eGFR, hs-CRP, nighttime sleep duration, Diabetes; Model3：Further adjusted for SBP at baseline.

**Table S16. Association between NC and risk of Hypertension by excluding CVD（N=42579）**

|  | low neck circumference | high neck circumference | *P* for trend |
| --- | --- | --- | --- |
| Model 1 | 1.000 | 1.163(1.122,1.207) | <0.001 |
| Model 2 | 1.000 | 1.099(1.059,1.141) | <0.001 |
| Model 3 | 1.000 | 1.060(1.021,1.100) | <0.001 |

Model 1：Adjusted for age and gender; Model 2：Further adjusted for Education level, Salt status, Smoking, Drinking , Physical activity, obesity,TC, eGFR, hs-CRP, nighttime sleep duration, Diabetes, Antidiabetic treatment, Lipid-lowering treatment; Model3：Further adjusted for SBP at baseline.

**Table S17. Association between NC and risk of Hypertension by excluding cancer（N=43148）**

|  | low neck circumference | high neck circumference | *P* for trend |
| --- | --- | --- | --- |
| Model 1 | 1.000 | 1.167(1.125,1.210) | <0.001 |
| Model 2 | 1.000 | 1.104(1.064,1.145) | <0.001 |
| Model 3 | 1.000 | 1.069(1.031,1.109) | <0.001 |

Model 1：Adjusted for age and gender; Model 2：Further adjusted for Education level, Salt status, Smoking, Drinking , Physical activity, obesity, TC, eGFR, hs-CRP, nighttime sleep duration, Diabetes, Antidiabetic treatment, Lipid-lowering treatment; Model3：Further adjusted for SBP at baseline.
